# COVID-19 Knowledge, Perception, Preventive Measures, Stigma, and Mental Health Among Healthcare Workers in Three Sub-Saharan African Countries: A Phone Survey

**DOI:** 10.4269/ajtmh.20-1621

**Published:** 2021-06-24

**Authors:** Nega Assefa, Abdramane Soura, Elena C. Hemler, Michelle L. Korte, Dongqing Wang, Yasir Y. Abdullahi, Bruno Lankoande, Ourohiré Millogo, Angela Chukwu, Firehiwot Workneh, Ali Sie, Yemane Berhane, Till Baernighausen, Ayoade Oduola, Wafaie W. Fawzi

**Affiliations:** 1 College of Health and Medical Sciences, Haramaya University, Harar, Ethiopia;; 2 Institut Supérieur des Sciences de la Population, University of Ouagadougou, Ouagadougou, Burkina Faso;; 3 Department of Global Health and Population, Harvard T.H. Chan School of Public Health, Harvard University, Boston, Massachusetts;; 4 Jegula Hospital, Harar, Ethiopia;; 5 Nouna Health Research Center, Nouna, Burkina Faso;; 6 Department of Statistics, University of Ibadan, Ibadan, Nigeria;; 7 Department of Epidemiology and Biostatics, Addis Continental Institute of Public Health, Addis Ababa, Ethiopia;; 8 Heidelberg Institute of Global Health, University of Heidelberg, Heidelberg, Germany;; 9 University of Ibadan Research Foundation, University of Ibadan, Ibadan, Nigeria;; 10 Department of Nutrition, Harvard T.H. Chan School of Public Health, Harvard University, Boston, Massachusetts;; 11 Department of Epidemiology, Harvard T.H. Chan School of Public Health, Harvard University, Boston, Massachusetts

## Abstract

The coronavirus disease 2019 (COVID-19) pandemic is an unprecedented public health crisis globally. Understanding healthcare providers’ (HCPs’) knowledge and perceptions of COVID-19 is crucial to identifying effective strategies to improve their ability to respond to the pandemic in sub-Saharan Africa. A phone-based survey of 900 HCPs in Burkina Faso, Ethiopia, and Nigeria (300 per country) was conducted to assess knowledge, perceptions, COVID-19 prevention measures, stigma, and mental health of HCPs. Modified Poisson regression models were used to evaluate predictors of knowledge, perceptions, and prevention measures; adjusted risk ratios (ARRs) and 95% confidence intervals (CIs) were calculated. Three-fourths of the HCPs had adequate knowledge, and over half had correct perceptions of risk and high levels of self-reported prevention measures. The majority of the HCPs (73.7%) reported self-perceived social stigma. There was relatively low prevalence of depression (6.6%), anxiety (6.6%), or psychological distress (18%). Compared with doctors, being a nurse was associated with lower levels of knowledge (ARR: 0.83; 95% CI: 0.77–0.90) and was also negatively associated with having correct perceptions toward COVID-19 (AOR: 0.82; 95% CI: 0.73–0.92). HCPs treating COVID-19 patients had higher likelihood of having high levels of prevention measures (AOR: 1.37; 95% CI: 1.23–1.53). Despite high levels of knowledge among HCPs in sub-Saharan Africa, there is a need to improve COVID-19 perceptions and compliance with prevention measures as well as address social stigma toward HCPs to better ensure their safety and prepare them to deliver health services.

## INTRODUCTION

The coronavirus disease 2019 (COVID-19) pandemic is an unmatched crisis and challenge for all nations.^1^ This highly infectious illness has exposed division, distrust, inequality, and trade tensions and, backed up by misinformation, spread across the world like a wildfire.^2,3^ The universal confusion from inaccurate information has been made worse by the speed of the progression and novelty of the virus, which has made it challenging to mitigate the consequences of the pandemic.^4,5^

COVID-19 is an impending danger for countries in sub-Saharan Africa (SSA) because its mitigation measures may have disastrous consequences given widespread poverty, fragile health systems, and a high prevalence of malnutrition, HIV, tuberculosis, and other comorbidities.^6,7^ At the beginning of the outbreak, many experts feared COVID-19 would be disastrous for SSA countries, though recent reports indicate that much of the continent has been spared these consequences.^8^ As of August 15, 2020, more than two million established COVID-19 cases and 50,000 deaths were reported on the continent, with 48,665 cases total in Nigeria, 27,242 cases in Ethiopia, and 1,237 in Burkina Faso.^9,10^ These figures account for a relatively small amount of global infections and an even smaller proportion of deaths.^11^ This could be the result of limited testing and reporting facilities. It may also reflect the relatively lower incorporation of some African countries in the world economy and the earlier prevention measures many African countries imposed.^12^

Healthcare providers (HCPs) are fighting at the forefront of the COVID-19 pandemic and are at risk due to potential exposure to COVID-19 patients. The International Council of Nurses determined that more than 230,000 HCPs were infected, and 600 nurses had lost their lives globally.^13^ The WHO reported that up to 100,000 HCPs in African countries might have been infected with COVID-19; however, this is likely underestimated.^14^

Poor knowledge of COVID-19 among HCPs leads to delayed diagnosis and shapes their perceptions of the pandemic, preventive measures taken, and infection control practices.^15^ For that reason, the WHO has issued several guidelines, online courses, and training to raise awareness, prevention, and control of COVID-19 among HCPs.^16,17^ Even though COVID-19 has offered some opportunities for HCPs to learn new skills, improve teamwork and team spirit, and increase handwashing and other preventive practices, it has also resulted in societal stigma and discrimination.^18^ Despite their well-deserved rewards, there are numerous reports of social stigma and isolation toward HCPs.^19^ There have been reports that HCPs practicing in informal settlements had trouble providing health services due to fear and stigmatization after COVID-19 exposure.^20^ Additionally, the psychological effects^18^ of the pandemic and mental health risks among HCPs are important issues to address.^19^

Very few studies have examined knowledge, prevention, and management of COVID-19 among HCPs. Understanding HCPs' COVID-19 knowledge, perceptions, and practices is crucial to identify effective strategies to contain the virus and safeguard HCPs’ physical and mental health in SSA. Identifying gaps in knowledge, perceptions, and preventive measures will help guide interventions to improve HCPs’ ability to respond to the pandemic. Therefore, using a novel mobile survey platform, this study aimed to explore the knowledge, perceptions, and preventive practices of HCPs toward COVID-19 prevention and management across three sub-Saharan African countries.

## MATERIALS AND METHODS

### Study settings.

This study was conducted in three sub-Saharan African countries: Burkina Faso, Ethiopia, and Nigeria. Healthcare workers, including nurses and physicians, were recruited in urban areas of each country (Ouagadougou in Burkina Faso, Addis Ababa in Ethiopia, and Ibadan and Lagos in Nigeria). The study rationale, sampling strategies, and the use of computer-assisted telephone interviewing technology in the study are described in detail elsewhere.^21^

### Study design.

This study was part of a planned repeated phone survey for HCPs in urban settings. The phone numbers of the HCPs were identified from lists provided by professional associations and health facilities in each country. Data collectors conducted interviews from virtual call centers using standardized electronic questionnaires. Professionals that exclusively practice medicine or nursing were recruited for the study. Each interview lasted 20–40 minutes. The study included 300 HCPs in each country (900 in total). From the overall lists of providers, we aimed to select 500 HCPs from each country to allow for a 60% nonresponse rate, but higher rates of nonresponse in some countries led us to select additional HCPs from the sampling frames in some sites to reach the target sample size.

This survey was approved by all necessary ethical review boards in each country, including the Harvard T.H. Chan School of Public Health Institutional Review Board, Nouna Health Research Center Ethical Committee and National Ethics Committee in Burkina Faso, the Institutional Ethical Review Board of Addis Continental Institute of Public Health in Ethiopia, and the University of Ibadan Research Ethics Committee and National Health Research Ethics Committee in Nigeria. All research staff members were trained on study procedures, including screening, consent, enrollment, and data collection, emphasizing confidentiality and safeguarding HCPs' rights and well-being. Experts translated the consent script and the surveys into the local languages of each respective country. The data collectors obtained verbal informed consent from each participant prior to beginning the interview.

### Data analysis.

The HCPs' responses were analyzed for each survey question by summarizing and computing scores within each country. The full questionnaire administered to HCPs is published elsewhere^21^ and included several sections. The knowledge section included 15 questions that all elicited a yes or no response about the pandemic's reality, symptoms, prevention, and control of COVID-19. The correct answer was counted as 1 point, and the incorrect/unknown answer was 0 points. Knowledge was analyzed at the correct item-by-item rate and a multi-dimensional knowledge score was calculated by summing up the items. The total points scored by each individual ranged between 0 and 15. Adequate knowledge toward COVID-19 was defined as a score ≥ of 13, moderate knowledge as a score between 7 and 12, and insufficient knowledge as a score of 6 or lower.^22–25^

The perception domain included 10 questions regarding facts and myths about COVID-19. The questions were also modified to suit the study area and settings appropriate for COVID-19. Four questions were related to the control of COVID-19, three questions were related to the transmission of the virus, one question was related to perceptions of preventive measures of COVID-19, and one question was related to treatment availability for COVID-19. A correct answer was counted as 1 point, and the incorrect/unknown answer got 0 points. The perceptions were first analyzed at the correct item-by-item rate. Then, the mean aggregated perception score was calculated by summing up the items. Each individual's total points ranged between 0 and 10, with a higher score indicating a correct perception toward COVID-19.^22–26^

The practice section was comprised of 10 questions about HCPs’ experience at the workplace since the onset of the pandemic in SSA. Questions were regarding provisions at the workplace that would prevent them from acquiring infection and workplace training, management, and handling options for suspected COVID-19 patients. All questions elicited yes or no answers. Correct answers were scored 1 point, and the incorrect/unknown answer was 0 points. Each individual's total score ranged between 0 and 10, with a high score indicating a high level of COVID-19 prevention measures.^23–26^

The questionnaire also assessed mental health and well-being, including social stigma and psychological stress.^27^ Psychological distress was measured over the past 2 weeks using the four-item Patient Health Questionnaire for Depression and Anxiety Scale (PHQ-4), with a total score ranging from 0 to 12. The scores of 2, 5, 8, and 9 are the cut-off points for none, mild, moderate, and severe psychological stress, respectively. The anxiety and depression subscales are the sums of the first and the last two questions of the PHQ-4, respectively, with a total score ranging from 0 to 6. On each subscale, a score of 3 or above was considered positive for anxiety and depression screening purposes.^27^

Descriptive analyses were conducted; means and SDs are presented for continuous variables, medians and interquartile ranges are presented for skewed variables, and counts and percentages are presented for categorical variables. Using the aggregated scores, knowledge, perception, and preventive practices were dichotomized. Factors associated with adequate knowledge, correct perceptions, and high level of prevention measures were identified by running modified Poisson regression models. Modified Poisson regression analysis was conducted using demographic characteristics including country of residence, facility managing authority, whether the HCP had treated COVID-19 patients, and availability of workplace guidelines as independent variables and adequate knowledge, correct perceptions, and high levels of prevention measures as outcome variables. Crude risk ratios (CRRs) and adjusted risk ratios (ARRs) were calculated with 95% confidence intervals (CIs). All data were managed and analyzed using Stata version 16. Variables that were significant in the univariate analysis (*P* < 0.2) were used to control for confounding for the final model. The level of statistical association used is *P* < 0.05.

## RESULTS

### Sociodemographic characteristics of the HCPs.

A total of 900 HCPs participated in this phone survey, with 300 from each country. Most participants provided nursing care (72% in Burkina Faso, 61% in Ethiopia, and 73% in Nigeria). Participants' average age was 40, 34, and 45 years, respectively, for Burkina Faso, Ethiopia, and Nigeria. Male healthcare providers accounted for around half of those surveyed in Burkina Faso (52%) and Ethiopia (47%), whereas they accounted for one-fifth in Nigeria. Approximately one-fifth of the HCPs in Burkina Faso worked in health outposts and clinics, whereas all participants in Ethiopia and Nigeria either worked in governmental or private hospitals (Table 1)

**Table 1:** Sociodemographic characteristics of healthcare providers in three sub-Saharan African countries

Sociodemographic characteristics	Burkina Faso	Ethiopia	Nigeria	Total
	Ouagadougou	Addis Ababa	Lagos and Ibadan	
Type of healthcare provided, *N* (%)				
Medicine	83 (27.67)	114 (38.00)	78 (26.00)	275 (30.56)
Nursing	217 (72.33)	183 (61.00)	220 (73.33)	620 (68.89)
Other^#^	0 (0)	3 (1.00)	2 (0.67)	5 (0.56)
Age, years, mean/median (SD/range)	39.73/37.50 (9.91/25–75)	34.40/30.00 (10.53/21–72)	45.18/45.00 (9.09/23–77)	39.77/39.00 (10.79/21–77)
Sex, *N* (%)				
Male	157 (52.33)	141 (47.00)	74 (24.67)	372 (41.33)
Female	143 (47.67)	159 (53.00)	226 (75.33)	528 (58.67)
Occupation, *N* (%)				
Doctor	81 (27.00)	120 (40.00)	77 (25.67)	278 (30.89)
Nurse and Other*	219 (73.00)	180 (60.00)	223 (74.00)	622 (69.11)
Facility, *N* (%)				
Government hospital/clinic	161 (53.67)	211 (70.33)	256 (85.33)	627(69.67)
Private hospital/clinic	71 (23.67)	89 (29.67)	44 (14.67)	205 (22.78)
Other^$^	68 (22.67)	0 (0)	0 (0)	68 (7.56)
Treated COVID-19 patients, *N* (%)				
Yes	41 (13.67)	192 (64.00)	130 (43.62)	365 (40.56)
No	259 (86.33)	108 (36.00)	168 (56.38)	535 (59.44)

HCP = healthcare provider; SD = standard deviation; $ = health outpost and clinics; * = clinical officers, community health workers; # = public health and surgery.

### Knowledge of COVID-19.

Most participants in Burkina Faso (95%), Ethiopia (77%), and Nigeria (99%) were concerned about the pandemic. Even though most of the survey participants recognized the major symptoms of COVID-19, fewer recognized skin rash as one of the symptoms. Almost all were knowledgeable about the methods of COVID-19 transmission. The aggregated mean knowledge scores (out of 15) in Burkina Faso, Ethiopia, and Nigeria were 12.97, 13.46, and 13.1, respectively (Table 2).

**Table 2:** COVID-19 knowledge among Healthcare Providers in three sub-Saharan African countries

Provider knowledge	Burkina Faso	Ethiopia	Nigeria	Total
	Ouagadougou	Addis Ababa	Lagos and Ibadan	
Concerned about COVID-19,* *N* (%)	284 (94.67)	230 (76.67)	297 (99.00)	811 (90.11)
Knew the main symptoms of COVID-19,* *N* (%)				
Weakness	273 (91.00)	295 (98.33)	290 (96.67)	858 (95.33)
Fever	293 (97.67)	297 (99.00)	292 (97.33)	882 (98.00)
Dry cough	292 (97.33)	298 (99.33	296 (98.67)	886 (98.44)
Knew less common symptoms of COVID-19*				
Sore throat	274 (91.33)	295 (98.33)	296 (98.67)	865 (96.11)
Headache	258 (86.00)	288 (96.00)	225 (75.00)	771 (85.67)
Runny nose	252 (84.00)	206 (68.67)	232 (77.33)	690 (76.67)
Skin rash	56 (18.6)	89 (29.67)	57 (19.00)	202 (22.44)
Muscle and joint aches	239 (79.67)	286 (95.33)	268 (89.33)	793 (88.11)
Loss of smell	237 (79.00)	269 (89.67)	293 (97.67)	799 (88.78)
Knew severe symptoms of COVID-19,* *N* (%)				
Shortness of breath	265 (88.33)	296 (98.67)	294 (98.00)	855 (95.00)
Knew transmission methods of COVID-19,* *N* (%)				
Respiratory droplets	293 (97.67)	296 (98.67)	291 (97.00)	880 (97.78)
Objects and surfaces	290 (96.67)	297 (99.00)	292 (97.33)	879 (97.67)
Physical contact	283 (94.33)	294 (98.00	292 (97.33)	869 (96.56)
Correctly identified false transmission methods of COVID-19,* *N* (%)				
Mosquito bites	294 (98.0)	244 (81.33)	273 (91.00)	811 (90.11)
Cellular mobile networks	292 (97.33)	290 (96.67)	240 (80.00)	822 (91.33)
Aggregated mean knowledge score (ranges: 0–15), mean (SD)	12.97 (1.69)	13.46 (1.19)	13.10 (1.20)	13.18 (1.39)
Total Mean knowledge score†	0.86 (0.11)	0.90 (0.08)	0.87 (0.08)	0.88 (0.09)
Knowledge status				
Adequate	213 (71.00)	248 (82.67)	218 (72.67)	679 (75.44)
Moderate/poor	87 (29.00)	52 (17.33)	82 (27.33)	221 (24.56)

* Number of observations (percentage).

† Mean/median (SD/range).

### Perceptions of COVID-19.

About 45%, 28%, and 29% of the participants in Burkina Faso, Ethiopia, and Nigeria, respectively, perceived a very high risk of contracting COVID-19, with 80% across all countries perceiving a high or very high risk. Almost all participants in each country had correct perceptions of prevention and transmission methods of COVID-19. Most HCPs in Burkina Faso believed “most COVID-19 patients die, but some survive,” whereas half of the Ethiopian and Nigerian HCPs did not believe so. The aggregated mean perception score in Burkina Faso was 9.38, compared with 8.43 in Ethiopia and 8.12 in Nigeria. Eighty-six percent of the HCPs in Burkina Faso had a correct perception level, compared with half in Ethiopia and 35% in Nigeria (Table 3).

**Table 3:** COVID-19 Perceptions of Healthcare Providers in three sub-Saharan African countries

Provider perceptions	Burkina Faso	Ethiopia	Nigeria	Total
	Ouagadougou	Addis Ababa	Lagos and Ibadan	
Perceived level of risk for COVID-19, *N* (%)				
No risk	1 (0.30)	4 (1.33)	4 (1.33)	9 (1.00)
Low risk	61(20.3)	51 (17.00)	42 (14.00)	154 (17.11)
High risk	104 (34.67	161 (53.67)	166 (53.33)	431 (47.89)
Very high	134 (44.67)	83 (27.67)	88 (29.33)	305 (33.89)
Ways to prevent transmission of COVID-19, *N* (%)				
Staying at home	271 (90.33)	298 (99.33)	270 (90.00)	839 (93.22)
Socially distancing	296 (98.67)	300 (100.00)	295 (98.33)	891 (99.00)
Regular washing hands	297 (99.00)	300 (100.00)	297 (99.00)	894 (99.33)
Sanitizing	297 (99.00)	300 (100.00)	294 (98.00)	891 (99.00)
Covering cough and sneeze	299 (99.67)	298 (99.33)	297 (99.00)	894 (99.33)
Wearing a mask	299(99.34)	296 (98.67)	298 (99.33)	892 (99.11)
Myths about ways to prevent COVID-19, *N* (% with correct answer)				
Sun exposure	278 (92.67)	182 (60.67)	199 (66.33	659 (73.22)
Drinking alcohol	295(98.83)	283 (94.33)	275 (91.67)	853 (94.78)
Lemon or ginger eat	276 (92.00)	167 (55.67)	130 (43.33)	573 (63.67)
Vitamin supplements	207 (69.00)	105 (35.00)	83 (27.67)	395 (43.89)
Severity of COVID-19, *N* (%)				
Everyone dies 2–4 weeks	1 (0.33)	6 (2.00)	0 (0.00)	7 (0.78)
Some survive, most die	3 (1.00)	23 (7.67)	51 (14.00)	77 (8.56)
Most survive, some die	274 (91.33)	244 (81.33)	213(53.33)	731 (81.22)
Almost everyone survives	21 (7.00)	26 (8.67)	24 (29.33)	71 (7.89)
Do not know/refusal	1 (0.33)	1 (0.33)	12 (4.00)	14 (1.55)
Aggregated mean perception score (range: 0–10)				
Mean (SD)	9.38 (0.82)	8.43 (1.13)	8.13 (1.19)	8.64 (1.18)
Total mean perception score, mean/median (SD/range)	0.94 (0.08)	0.84 (0.11)	0.81 (0.12)	0.86 (0.12)
Perception status, *N* (%)				
Positive	257 (85.67)	150 (50.00)	104 (34.67)	511 (56.78)
Negative	43 (14.33)	150 (50.00)	196 (65.33)	389 (43.22)

### COVID-19 prevention measures.

Nearly all participants self-reported regularly wearing masks, using sanitizers and washing stations, washing their hands, and cleaning and decontaminating in their respective facilities, and most reported wearing personal protective equipment. A significant portion of the HCPs (73%) in Nigeria had previously treated COVID-19 patients at their workplace, compared with one-fifth of the HCPs in Burkina Faso and half of the HCPs in Ethiopia. Eighty-three percent of the HCPs in Burkina Faso, 61% in Ethiopia, and 98% in Nigeria had workplace guidelines for COVID-19. Most participants (62% in Burkina Faso, 68% in Ethiopia, and 89% in Nigeria) received training on the disease's natural course. Healthcare providers in Nigeria had higher aggregated mean levels of COVID-19 prevention measures compared with Burkina Faso and Ethiopia. In Nigeria, 82% had high levels of prevention measures, compared with 50% in Ethiopia and 39% in Burkina Faso (Table 4).

**Table 4:** Preventive measures available in the workplaces of healthcare providers in three sub-Saharan African countries

		Burkina Faso	Ethiopia	Nigeria	Total
		Ouagadougou	Addis Ababa	Lagos and Ibadan	
COVID-19 Preventive measures*	Wearing mask	300 (100)	298 (99.33)	295 (98.33)	893 (99.22)
	Wearing PPE	255 (85.00)	267 (89.00)	250 (83.33)	772 (85.78)
	Handwashing with water and soap	296 (98.67)	293 (97.67)	298 (99.33)	887 (98.56)
	Sufficient distance between patients	276 (92.00)	246 (82.00)	280 (93.33)	802 (89.11)
	Presence of sanitizers and handwashing station	287 (95.67)	278 (92.67)	297 (99.00)	862 (95.78)
	Regular cleaning and decontamination	276 (92.00)	282 (94.0)	293 (9767)	851 (94.56)
COVID-19 training or formal orientation COVID-19*	The natural course of COVID-19 disease	187 (62.33)	203 (67.67)	266 (88.67)	656 (72.89)
	Management and treatment of the disease,	133 (44.33)	201 (67.00)	258 (86.00)	592 (65.78)
	Management health conditions with COVID-19	130 (43.33)	175 (58.33)	257 (85.67)	562 (62.44)
	Available options for suspected patients of COVID-19	150 (50.00)	182 (60.67)	259 (86.33)	599 (66.56)
Presence of guidance on COVID-19*		249 (83.0)	182 (60.67)	295 (98.33)	726 (80.67)
Action to be taken if COVID-19 diagnosed*	Refer to another facility	185 (74.30)‡	67 (36.81)§	79 (26.28)ǁ	331 (45.59)
	Treat at the facility	60 (24.10)	93 (51.10)	216 (73.22)	369 (50.83)
	Send home	4 (1.61)	22 (12.09)	0 (0)	26 (3.58)
Preventive measure score	Range: 0 to 10	7.63 (1.92)	8.11 (2.06)	9.18 (1.42)	8.31 (1.93)
Mean preventive measure score†	Total mean	0.80 (0.17)	0.84 (0.18)	0.92 (0.12)	0.85 (0.17)
Preventive measure status*	High	118 (39.33)	167 (55.67)	246 (82.00)	531 (59.00)
	Low	182 (60.67)	133 (44.33)	54 (18.00)	369 (41.00)

PPE = personal protective equipment.

SD = standard deviation.

* Number of observations (percentage).

† Mean/median (SD/range).

‡ 249 observations.

§ 182 observations.

ǁ 295 observations.

### Mental health and well-being.

The majority of the participants (88.4%) reported they did not drink alcohol, and 64.1% reported no change in sleeping habits since the start of the pandemic. Most HCPs in Burkina Faso (59%) and Nigeria (86%) experienced social acknowledgment, whereas only 25% in Ethiopia did. A smaller proportion of Ethiopian participants (1.7%) reported violence against HCPs, whereas 5% and 7% of the participants in Burkina Faso and Nigeria did. Even though only a small proportion of participants reported physical violence and service denial, most perceived social stigma toward HCPs, with 88% of HCPs in Ethiopia reporting social stigma. Based on the aggregated psychological stress score, 82% had no psychological distress (Table 5).

**Table 5:** Healthcare provider mental health and wellbeing during COVID-19 in three sub-Saharan African countries

		Burkina Faso	Ethiopia	Nigeria	Total
		Ouagadougou	Addis Ababa	Lagos and Ibadan	
Alcohol drinking habits*	Do not drink alcohol	229 (76.33)	238 (79.30)	284 (94.67)	751 (88.44)
	Drank less	33(11.00)	38 (12.67)	12 (4.00)	83 (9.22)
	Drank same	36 (12.00)	19 (6.33)	4 (1.33)	59 (6.56)
	Drank more	2 (0.67)	5 (1.67)	0 (0)	7 (0.78)
Sleeping habits*	Sleep less	97 (32.33)	40 (13.33)	49 (16.33)	186 (20.67)
	Sleep the same	176 (58.67)	202 (67.33)	198 (66.00)	577 (64.11)
	Sleep better	27 (9.00)	57 (17.67)	53 (17.67)	137 (15.22)
Perceived stigma*	Social avoidance or rejection	108 (35.88)	131 (43.67)	175 (58.33)	414 (46.00)
	Denial of services	9 (2.99)	45 (15.00)	11 (3.67)	65 (7.22)
	Physical violence	16 (5.32)	5 (1.67)	22 (7.33)	43 (4.78)
	No Acknowledgment	123 (41.00)	225 (75.00)	42 (14.00)	390 (43.33)
	Perceived Stigma (aggregated)	198 (66.00)	263 (87.67)	202 (67.33)	663 (73.67)
Feeling nervous, anxious, or on edge*	Not at all	215 (71.17)	256 (85.33)	217 (72.33)	688 (76.44)
	Several days	26 (8.67)	17 (5.67)	42 (14.00)	85 (9.44)
	More than half the days	44(14.67)	18 (6.00)	36 (12.00)	98 (10.89)
	Nearly every day	15 (5.00)	9 (3.00)	5 (1.67)	29 (3.22)
Not being able to halt or control worrying*	Not at all	235(78.33)	272 (90.67)	231 (77.00)	738 (82.00)
	Several days	18 (6.00)	10 (3.33)	36 (12.00)	64 (7.11)
	More than half the days	38 (12.67)	15 (5.00)	30 (10.00)	83 (9.22)
	Nearly every day	9 (3.00)	3 (1.00)	3 (1.00)	15 (1.67)
Feeling down, depressed or hopeless*	Not at all	233 (77.67)	278 (92.67)	262 (87.33)	773 (85.89)
	Several days	20 (6.67)	12 (4.00)	28 (9.33)	60 (6.67)
	More than half the days	37 (12.33)	7 (2.33)	8 (2.67)	52 (5.78)
	Nearly every day	10 (3.33)	3 (1.00)	2 (0.67)	15 (1.67)
Little interest or pleasure in doing things*	Not at all	272 (90.7)	266 (88.67)	269 (89.67)	807 (89.67)
	Several days	6 (2.00)	18 (6.00)	23 (7.67)	47 (5.22)
	More than half the days	17 (5.67)	5 (1.67)	4 (1.33)	26 (2.89)
	Nearly every day	5 (1.67)	11 (3.67)	4 (1.33)	20 (2.22)
Psychological distress score†	Total score: 12	1.53 (2.34)	0.75 (1.76)	1.09 (1.97)	1.12 (2.06)
Anxiety*		40 (13.33)	6 (2.00)	13 (4.33)	59 (6.56)
Depression*		29 (9.67)	18 (6.00)	12 (4.00)	59 (6.56)
Psychological distress*	None	232 (77.33)	262 (87.33)	243 (81.00)	737 (81.89)
	Mild	41 (13.67)	30 (10.00)	46 (15.33)	117 (13.00)
	Moderate	23 (7.67)	5 (1.67)	8 (2.67)	36 (4.00)
	Severe	4 (1.33)	3 (1.00)	3 (1.00)	10 (1.11)

SD = standard deviation.

* Number of observations (percentage).

† Mean/median (SD/range).

### Factors associated with adequate knowledge of COVID-19 among HCPs.

Ethiopian HCPs were 12% more likely (ARR: 1.12; 95% CI: 1.02–1.25) to have adequate knowledge of COVID-19 compared with HCPs from Burkina Faso. Compared with doctors, nursing practitioners were 17% less likely to have adequate COVID-19 knowledge (ARR: 0.83; 95% CI: 0.77–0.90). Healthcare poviders working in government facilities were not different from those working at private clinics with respect to having adequate knowledge. Similarly, there was no difference between male and female HCPs, and age was not a significant predictor of knowledge (Table 6).

**Table 6:** Factors associated with adequate COVID-19 knowledge among healthcare providers in three sub-Saharan African countries

	CRR	95% CI	*P* value	ARR	95% CI	*P* value
Country						
Burkina Faso	ref			ref		
Ethiopia	1.16	1.10–2.76	0.023**	1.12	1.02–1.25	0.027**
Nigeria	1.02	0.73–1.69	0.62	1.02	0.91–1.14	0.74
Sex						
Male	1.03	0.95–1.11	0.49	0.97	0.89–1.05	0.44
Female	ref			ref		
Age, year	0.99	0.99–1.00	0.17	1.00	0.99–1.00	0.98
Occupation						
Doctors	ref			ref		
Nurses & Other	0.44	0.30–0.65	0.000**	0.83	0.77–0.90	0.000**
Facility						
Government	1.11	0.94–1.31	0.22	1.03	0.86–1.24	0.74
Private	1.07	0.90–1.28	0.44	0.961	0.79–1.16	0.64
Others	ref			ref		
Treated COVID-19 patients						
Yes	1.04	0.97–1.12	0.81	0.99	0.91–1.07	0.73
No	ref			ref		
Workplace guidelines						
Yes	0.91	0.83−0.98	0.036	0.93	0.85−1.02	0.14
No	ref			ref		

CRR = crude risk ratio; ARR = adjusted risk ratio.

** Significant at *P* < 0.005.

### Factors associated with a correct perception of COVID-19 among HCPs.

Most variables had a statistically significant association with correct perceptions of the COVID-19 pandemic in the univariate analysis. Compared with the HCPs in Burkina Faso, those in Ethiopia (ARR: 0.57; 95% CI: 0.49–0.66) and Nigeria (ARR: 0.43; 95% CI: 0.36–0.51) were less likely to have correct perceptions of COVID-19. Nurses and other HCPs were 18% less likely (ARR: 0.82; 95% CI: 0.73–0.92) to have correct perceptions compared with doctors. In adjusted models, HCPs who had treated COVID-19 patients had no difference in risk (ARR: 1.02; 95% CI: 0.89–1.17) for correct perception compared with those who had not treated COVID-19 patients. Healthcare providers with workplace guidelines were 10% less likely to have correct perception than their counterparts, but this relationship was not statistically significant (ARR: 0.90; 95% CI: 0.79–1.02). All variables, except country and occupation, were not statistically significantly associated with correct perception (Table 7).

**Table 7:** Factors associated with correct perceptions towards COVID-19 among healthcare providers in three sub-Saharan African countries

	CRR	95% CI	*P* value	ARR	95% CI	*P* value
Country						
Burkina Faso	ref			ref		
Ethiopia	0.15	0.09–0.24	0.000**	0.57	0.49–0.66	0.000**
Nigeria	0.10	0.0−0.16	0.000**	0.43	0.36−0.51	0.000**
Sex						
Male	1.34	1.20–1.50	0.000**	1.09	0.98-1.22	0.10
Female	ref	ref		ref	ref	
Age, year	0.99	0.99–1.00	0.81	1.00	0.99-1.01	0.98
Occupation						
Doctors	ref			ref		
Nurses & Other	0.81	0.73–0.91	0.001**	0.82	0.73–0.92	0.001**
Facility						
Government	0.59	0.53–0.66	0.000**	0.94	0.85–1.05	0.31
Private	0.68	0.59–0.78	0.00**	0.94	0.82–1.08	0.38
Others	ref			ref		
Treated COVID-19 patients						
Yes	1.04	0.76–0.98	0.02**	1.02	0.89–1.17	0.81
No	ref			ref		
Workplace guidelines						
Yes	0.86	0.76–0.98	0.03**	0.90	0.79–1.02	0.11
No	ref			ref		
Perceived stigma						
Yes	0.81	0.57–1.16	0.25	0.92	0.84–1.04	0.20
No	ref			ref		

CRR = crude risk ratio; ARR = adjusted risk ratio.

** Significant at *P* < 0.005.

### Factors associated with high levels of COVID-19 prevention measures among HCPs.

In the final analysis, factors associated with high COVID-19 preventive measures were country, treating COVID-19 patients, and the presence of workplace guidelines. Study participants were more likely to have high levels of prevention measures than their counterparts if they had treated COVID-19 patients (ARR: 1.37; 95% CI: 1.23–1.53) or had guidelines regarding COVID-19 in their workplace (ARR: 1.65; 95% CI: 1.34–2.02). Healthcare providers working in governmental institutions were 21% more likely (ARR: 1.21; 95% CI: 0.84–1.75) to have high levels of prevention measures than those working in health outposts and clinics. Healthcare providers in Nigeria were 78% more likely to have high preventive measures (ARR 1.78; 95% CI: 1.49–2.12) compared with those in Burkina Faso (Table 8).

**Table 8:** Factors associated with high levels of prevention measures of healthcare providers towards COVID-19 in three sub-Saharan African countries

	CRR	95% CI	*P* value	ARR	95% CI	*P* value
Country						
Burkina Faso	ref			ref		
Ethiopia	1.42	1.19–1.68	0.000**	1.29	1.06–1.56	0.011**
Nigeria	2.08	1.79−2.41	0.000**	1.78	1.49−2.12	0.000**
Sex						
Male	0.97	0.87–1.09	0.63	NA	NA	NA
Female	ref			ref		
Age, year	1.00	0.99–1.01	0.19	1.00	0.99–1.00	0.13
Occupation						
Doctors	ref			ref		
Nurses & Other	0.97	0.87–1.09	0.66	NA	NA	NA
Facility						
Government	1.98	1.42−2.78	0.000**	1.21	0.84−1.75	0.29
Private	0.65	0.87–1.81	0.23	0.91	0.62–1.33	0.63
Others	ref			ref		
Treated COVID-19 patients						
Yes	1.54	1.38−1.71	0.000**	1.37	1.23−1.53	0.000**
No	ref			ref		
Workplace guidelines						
Yes	1.85	1.50−2.27	0.000**	1.65	1.34−2.02	0.000**
No	ref			ref		
Adequate Knowledge						
Yes	1.03	0.91−1.18	0.58	1.03	0.92−1.16	0.58
No	ref			ref		
Correct Perception						
Yes	0.84	0.76−0.94	0.002**	1.10	0.99–1.22	0.06
No	ref			ref		

CRR = crude risk ratio; ARR = adjusted risk ratio; NA = could not be computed.

** Significant at *P* < 0.005.

## DISCUSSION

We found that about three-fourths of HCPs had an adequate level of knowledge, and more than half had correct perceptions and high levels of prevention measures related to COVID-19. The majority of the HCPs perceived social stigma due to COVID-19. However, most were not experiencing depression, anxiety, or psychological distress. Occupation was a predictor of having adequate knowledge of COVID-19. The HCPs’ country was associated with both adequate knowledge and correct perceptions; the participant's occupation was also associated with correct perceptions toward COVID-19. Healthcare providers with experience treating COVID-19 patients had higher levels of COVID-19 prevention measures.

Most participants could correctly identify the most common and severe symptoms of COVID-19 and the virus's transmission methods. However, fewer participants could identify skin rash and runny nose as less common symptoms of COVID-19. This finding highlights that health authorities should continue to encourage HCPs to access information from reliable sources and educational courses focusing on less common presentation and management of COVID-19.^28^ Even though participants practicing nursing were less likely to be knowledgeable than physicians, the average knowledge score was still 87%, indicating a high level of COVID-19 knowledge among HCPs. The high level of knowledge could be due to a high proportion of HCPs (73%) having received training and scientific information on COVID-19. In contrast, a web-based survey in India reported that most HCPs (61%) used social media as a source of information and found that HCPs had poor knowledge about COVID-19 transmission and symptom onset, although they had positive perceptions of COVID-19 prevention and control.^29^ Factors associated with insufficient knowledge and perceptions in this study were age and profession. Another multicenter study in Ethiopia reported that many HCPs had adequate knowledge and correct perceptions of COVID-19 and that using social media, telecommunication, and television/radio were significantly associated with higher knowledge.^30^ In addition, an online cross-sectional study conducted in Uganda identified factors such as higher age and consumption of news media that were significantly associated with knowledge.^31^

We found that around half of the HCPs had correct perceptions of COVID-19 and that 34% of the participants thought they were at very high risk of infection. Almost all of the respondents were willing to wash and sanitize their hands regularly, practice social distancing, and use facemasks to control the pandemic's transmission. These results suggest that most of the respondents were confident in protecting themselves and were aware that COVID-19 is very contagious. Even though about 73.2%, 95%, and 64% of the HCPs correctly answered that sunbathing, drinking alcohol or tea, and ginger were not strategies to relieve the symptoms of COVID-19, more than half (56.1%) of them chose vitamin supplementation as a preventive strategy. Although vitamins and supplements boost the immune system for fighting any infection, there is no evidence in their efficacy for treating COVID-19.^32,33^ Therefore, regular updating of scientific information through the official webpages of WHO and health ministries of the respective country is needed to provide HCPs with the most up-to-date scientific information.^28^

Even though most HCPs reported wearing masks, washing their hands, and having sufficient sanitizers and washing stations at their workplace, only three-fifths had high levels of COVID-19 prevention measures. The HCPs who worked in a facility with COVID-19 workplace guidelines and cared for COVID-19 patients were more likely to practice high levels of prevention measures than their counterparts. In other studies, the type of facility was important: for instance, HCPs working in governmental facilities in China were more likely to have guidelines or policies and treat COVID-19 with an adequate supply of personal protective equipment.^15,34^ However, health workers are at great risk of infection, as noted in a study from the United Kingdom that estimated the risk of contracting COVID-19 in frontline HCPs was 12 times more than the general community,^35^ highlighting the importance of continued training and provision of preventive supplies to keep frontline workers safe.

Even though HCPs reported the same sleeping pattern since the pandemic, about 46% and 43% experienced social avoidance and disapproval. Most of the HCPs perceived social stigma due to COVID-19. However, less than one-fifth showed signs of psychological distress, and only 7% scored positive for anxiety and depression. Although psychological morbidity was low in this study, psychological morbidity has been reported as higher in India and China; therefore, programs and policies are needed to keep the morbidity low.^36,37^ These results could reflect the smaller toll of the pandemic in SSA compared with other places.

The use of data from multiple sites across SSA and the use of phone-based surveys to enable remote data collection during the pandemic to avail fresh information are strengths of this study. It is important to note that the information collected in this study is from major urban areas of the countries included. Hence, it is uncertain whether the results may be generalizable to health care workers in each country overall, especially to those in rural areas. Furthermore, this study was a phone-based survey and therefore has the potential for introduction of selection bias because the report is based on only voluntary respondents.

Even though most HCPs in Burkina Faso, Ethiopia, and Nigeria had adequate knowledge of COVID-19, there is a need to improve COVID-19 perceptions and compliance with prevention measures. The level of knowledge, perception, and prevention measures regarding COVID-19 may differ in each country due to the caseload, epidemic curve, and the death toll in each country. An intersectoral approach is needed to combat COVID-19 in SSA to increase compliance with prevention measures and correct perceptions toward the virus. Due to the relatively moderate sample size and the cross-sectional design, further large-scale and longitudinal surveys or interventional studies are needed to understand further the long-term impacts of the COVID-19 pandemic on HCPs and the health systems in SSA. Because COVID-19 and its management are new phenomena to the world, potential factors that would affect knowledge acquisition and translation of knowledge to practices need to be studied from each country’s perspective.
